# Impact of taxes on purchases of close substitute foods: analysis of cross-price elasticities using data from a randomized experiment

**DOI:** 10.1186/s12937-021-00736-y

**Published:** 2021-09-07

**Authors:** Jody C. Hoenink, Wilma E. Waterlander, Joreintje D. Mackenbach, Cliona Ni Mhurchu, Nick Wilson, Joline W. J. Beulens, Nhung Nghiem

**Affiliations:** 1 Amsterdam UMC, Vrije Universiteit Amsterdam, Department of Epidemiology and Data Science, Amsterdam Public Health Research Institute, De Boelelaan 1089a, 1081 HV Amsterdam, the Netherlands; 2 Upstream Team, www.upstreamteam.nl, Amsterdam UMC, Amsterdam, The Netherlands; 3 Amsterdam UMC, Department of Public and Occupational Health, University of Amsterdam, Amsterdam Public Health Research Institute, Meibergdreef 9, Amsterdam, the Netherlands; 4 National Institute for Health Innovation, University of Auckland, Auckland, New Zealand; 5 Department of Public Health, University of Otago, Wellington, New Zealand; 6 Julius Center for Health Sciences and Primary Care, University Medical Center Utrecht, Utrecht University, Utrecht, the Netherlands

**Keywords:** Pricing strategies, Intervention study, Food policy, Food taxes

## Abstract

**Objective:**

To examine the effects of health-related food taxes on substitution and complementary purchases *within* food groups, including from unhealthier to healthier alternatives and between brands.

**Methods:**

We used data from a virtual supermarket experiment with data from 4,259 shopping events linked to varying price sets. Substitution or complementary effects within six frequently purchased food categories were analyzed. Products’ own- and cross-price elasticities were analyzed using Almost Ideal Demand System models.

**Results:**

Overall, 37.5% of cross-price elasticities were significant (*p* < 0.05) and included values greater than 0.10. Supplementary and complementary effects were particularly found in the dairy, meats and snacks categories. For example, a 1% increase in the price of high saturated fat dairy was associated with a 0.18% (SE 0.06%) increase in purchases of low saturated fat dairy. For name- and home-brand products, significant substitution effects were found in 50% (n = 3) of cases, but only in one case this was above the 0.10 threshold.

**Conclusions/policy implications:**

Given the relatively low own-price elasticities and the limited substitution and complementary effects, relatively high taxes are needed to substantively increase healthy food purchases at the population level.

**Trial registration:**

This study included secondary analyses; the original trial was registered in the Australian New Zealand Clinical Trials Registry ACTRN12616000122459.

**Supplementary Information:**

The online version contains supplementary material available at 10.1186/s12937-021-00736-y.

## Introduction

A suboptimal diet is an important preventable risk factor for non-communicable diseases (NCDs) [1]. For example, high consumption of sodium and sugar sweetened beverages is associated with cardiovascular disease [2] and type 2 diabetes [3]. A systematic review investigating different intervention strategies found that health-related fiscal interventions showed the most promise in improving diets [4]. Studies investigating the effect of health-related taxes and subsidies have generally found that subsidies on healthy foods effectively increase purchases of targeted products, and taxes on unhealthy foods decrease purchases of targeted products [4, 5]. However, these effects may not necessarily translate into healthier diets. For example, while studies generally find that a sugar sweetened beverages (SSB) tax decreases SSB purchases [6, 7], the effect of taxes on diet may be weakened if the taxed foods and beverages are replaced by other untaxed or cheaper unhealthy foods and beverages (hereafter referred to as foods) such as home-brand products [8].

Substitution effects are an important determinant of the ultimate impact of health-related taxes and subsidies [9]. For example, the Price ExaM Study examined the effect of subsidies and taxes on food purchasing behavior and found that a saturated fat tax resulted in a 16.2% (95%CI -18.8; -13.6) decrease of saturated fat, but also a 5.0% (95%CI 2.1; 7.9) increase of sugar as a percentage of total energy purchased [10]. These substitution effects can be quantified using cross-price elasticity values. Positive cross-price elasticities indicate that foods are substitutes and negative cross-price elasticities indicate that foods are complements [11] and cross-price elasticities are likely to be larger when there are close substitutes/complements for a certain food (e.g., within the same food group) [12].

Currently, adequate data for the estimation of cross-price elasticities is lacking [5, 13]. When studies include cross-price elasticities, reported food groups are often highly aggregated (e.g., all soft drinks) as opposed to the level of disaggregation that is required to study detailed substitution effects (e.g., from regular soft drinks to diet soft drinks) [14]. The high level of aggregation usually arises because most studies use nutrition survey data to estimate price elasticities (i.e., modelling studies), which often do not include the level of detail needed to sufficiently estimate the price elasticities of, for example, name- and home-brand products [15]. Using empirical purchasing data provides a unique opportunity to construct the disaggregated food groups needed to be able to estimate price elasticities for smaller food groups.

Experimental studies in validated virtual supermarket environments allow for the measurement of own- and cross-price elasticities for food groups of interest before policies are implemented in real-world settings [16]. The aim of this study was to examine the substitution and complementary effects of health-related food and beverage taxes within food groups, including from unhealthier to healthier alternatives and between different brand alternatives. Our hypothesis was that if the price of products high in sugar, sodium and saturated fat would increase, individuals would substitute these products with healthier alternatives within that same food group. Also, we expected that home-brand products were substitutes for name-brand products.

## Methods

We used data from the Price Experiment and Modelling (Price ExaM) Study. A study protocol for the Price ExaM Study, including a full description of the experiment and modelling methods, has been published elsewhere [17] as well as the overall results of the Price ExaM study [10, 11]. The Price ExaM Study was an experimental study conducted in 2016 in a virtual supermarket (VS) setting where participants were exposed to random price variations simulating an average New Zealand supermarket (the control price set), a fruit and vegetable subsidy, an SSB tax, a saturated fat tax, a salt tax, or a sugar tax. Full details about this study can be found elsewhere [10, 17], but a brief description is provided below.

### Price ExaM Study

The main aim of the Price ExaM Study was to provide high quality evidence on the impact of health-related food taxes and subsidies by estimating precise and accurate own-price and cross-price elasticities [17]. For this, 5000 different price sets were created with random price variations for all 1411 food and beverage products within the VS [17]. In addition to including random price variations, the price sets also included systematic price variations for foods and beverages to simulate several subsidy and taxing policy scenarios, including a SSB tax (at either 20% or 40%), a saturated fat tax (NZ$2 per 100 g and NZ$4 per 100 g), a salt tax (NZ$0.02 per 100 mg; equivalent to NZ$0.04 per 100 mg sodium), a sugar tax (NZ$0.20 per 100 g and NZ$0.40 per 100 g), and a 20% fruit and vegetable subsidy. Some price sets included two or more tax and subsidy options affecting food prices.

From February 2016 to December 2016, 2352 participants were registered in the study. In total, 1132 participants were randomly assigned to the different price sets in the VS. Mean age of participants was 32.9 years (SD 12.5), 79.2% were female, 67% had completed tertiary level education, and 71.3% were New Zealand European [10]. Overall, 743 (71.6%) completed the study (i.e., conducted all five shops). The Price ExaM Study was approved by the University of Auckland Human Participants Ethics Committee (reference 016,151) [17].

### Data preparation for the current study

From the Price ExaM Study, we included data available from all 4259 shopping events; including price variations and correlating shopping patterns of 18 food categories. From this dataset, those food categories including products that were frequently purchased were selected, this included six food categories: beverages, grains, dairy, meat, sauces and snacks (including desserts). Fruit and vegetables were excluded because all products in this category are generally healthy and therefore the substitution effects within these groups are not of great interest from a public health perspective. All food categories were disaggregated into smaller food groups based on their sugar, sodium and saturated fat (SAFA) content. Cut-off values for low, medium or high levels of sugar/sodium/SAFA were based on the traffic light label threshold guidelines of the United Kingdom, which can be applied to all types of foods and non-alcoholic beverages [18, 19]. Food categories were only disaggregated into the smaller nutritional clusters when these categories included products within all three levels of sugar, sodium and/or SAFA. An example of a nutritional cluster is dairy foods with low, medium and high levels of sodium and SAFA. The food category dairy was not further disaggregated into groups of products with varying levels of sugar as no dairy products fell into the high-sugar category. Supplementary Table 1 displays the different food groups and their cut-off points and Supplementary Tables 2a and b display the food items found within the nutritional clusters.

In order to assess the overall healthiness of purchases, foods were categorized as healthy or unhealthy. This is important as it is possible for foods to contain a low amount of one adverse nutrient (e.g. sugar) but a high amount of another nutrient (e.g. sodium), meaning that such foods are not necessarily healthier overall. Fresh fish and packaged foods eligible to carry a health claim based on the New Zealand and Australian government-endorsed nutrient profiling system (Nutrient Profiling Scoring Criterion [20]) were classified as healthy. All other foods were classified as unhealthy. Supplementary Table 3 displays the nutrient content and the percentage of products classified as healthy within the nutritional clusters. In all cases but one, low sugar/sodium/SAFA clusters included more healthy products compared to medium or high sugar/sodium/SAFA clusters. Also, nutritional clusters high in sugar/sodium/SAFA included far less healthy products compared to nutritional clusters with medium levels of sugar/sodium/SAFA. For beverages, grains, dairy and meats, the sugar/sodium/SAFA nutrients seem to cluster together, e.g., medium and high sugar beverages also contain relatively high amounts of sodium and medium and high sodium dairy also contain high amounts of saturated fat.

For name- and home-brand food groups, food categories with at least 20 home-brand products were selected. The resulting food categories that were divided into name- and home-brand food groups included beverages, grains and snacks. The name- and home-brand food groups generally included a similar percentage of healthy products, with the exception of grains where 59.7% of name-brand products were classified as healthy compared to 80.0% of home-brand products (Supplementary Table 3).

### Data analyses

#### The Almost Ideal Demand System model

Using price elasticities, we can determine the percentage change in the demand for product X if its own price changes (own-price elasticity) or if the price of other products (Y, Z) changes (cross-price elasticity) [21]. Typically, items that are consumed together (complementary products) have a negative cross-elasticity, while items that can be substituted (e.g., coffee for tea) have a positive cross-elasticity. In this study, substitution and complementary effects were examined using uncompensated cross-price elasticities modelled by the Almost Ideal Demand System (AIDS) [22]. Uncompensated price elasticities estimate the impact of a price increase on food purchases when consumers’ money income is held constant [23]. Analysis was at the level of the household, not the individual, as participants in the virtual supermarket conducted shopping events for their entire household. Analyses were conducted using the package ‘quaids’ by Poi in STATA version 15.0 [24]. The package ‘quaids’ is a user-friendly and widely used package (e.g., [25–27]) that allows researchers to fit the AIDS model without writing their own program and to adjust for demographic variables and clustered data. Using a validated econometrics package helps with model quality control as well. Censored data are usually not a problem within the ‘quaids’ model when analyzing data from aggregated food groups. However, given that estimations within the package only run at a minimum of three goods within disaggregated food groups, this presented a larger problem. While data on all 4259 shopping events were used to estimate the price elasticities (i.e., no distinction between the different taxing policy scenarios were made), the data was censored as zero-purchasers were excluded from the analyses; only shopping events where participants purchased at least one product in each nutritional cluster were included (e.g., only shops with products purchased from low, medium and high nutritional clusters within the dairy category). This led to each AIDS model consisting of different numbers of shopping events. Nevertheless, the AIDS model estimated by the ‘quaids’ package was preferred over other models to calculate cross-price elasticities as it satisfies micro-economics restrictions such as adding-up and allowed for the estimation of uncompensated (Marshallian) elasticities. Uncompensated price elasticities are most commonly reported in studies and are arguably most relevant for policy [28].

In total, N = 12 AIDS models were run, leading to a total of N = 36 own-price elasticities and N = 72 cross-price elasticities across all food groups. Although we used data from a randomized experiment, our models were adjusted for age, sex, highest attained educational level, ethnicity of the main shopper and household size because the number of participants in certain arms were low [10]. Statistical significance was set at a p-value of < 0.05 and a relevant effect size for cross-price elasticities was set at cross-price elasticities ≥ 0.10. Results regarding the expenditure and compensated price elasticities can be found in the Supplementary Material.

#### The double log model

AIDS models with only two groups are reduced to only one equation to be estimated. Given the microeconomic restrictions such as adding-up and symmetry on the estimated parameters, the one equation will be reduced to a very strict functional form and hence can produce unreliable estimates [22]. Therefore, the double log model was used to calculate price elasticities for name- and home-brand products within each aggregate food category [29]. Linear mixed models with the quantity of name- or home-brand products sold within each food category were used as the dependent variable. The independent variables included the prices of the name- and home-brand products and demographic variables. In order to calculate own- and cross-price elasticities, the standard log–log functional form of the dependent and independent variables was applied, as was done in this previous study [29].

## Results

**Fig. 1:**
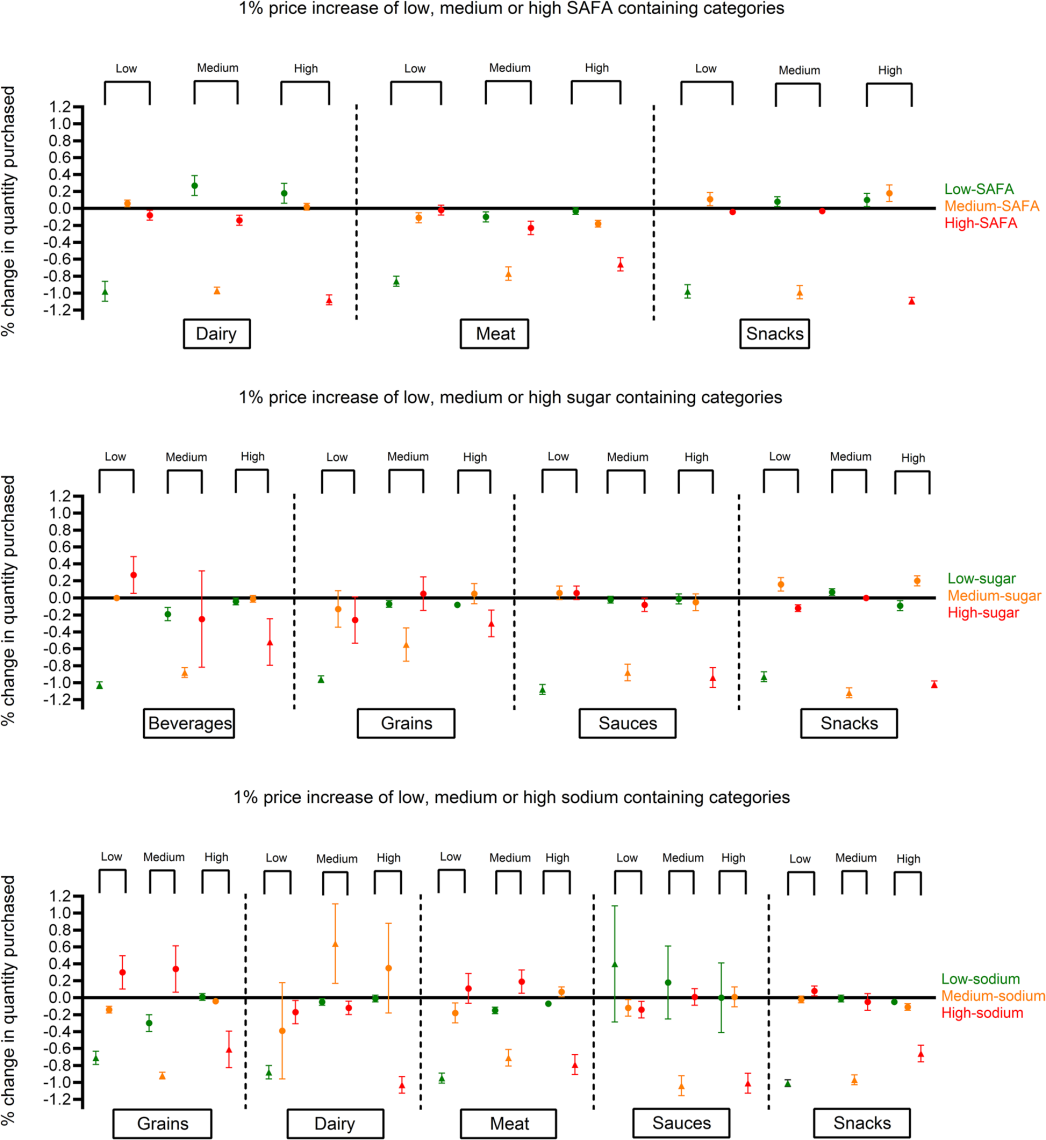
Change in demand (%) as a response to a 1% price increase of low, medium or high SAFA/sugar/salt containing categories adjusted for demographic variables. Uncompensated own-price elasticities are displayed as triangles and uncompensated cross-price elasticities are displayed as dots. Dots above the line represent substitution effects and dots below the line represent complementary effects

Tables 1 and 2 show the data used to estimate the price elasticities for the nutritional clusters and name- and home-brand food groups (Supplementary Table 4 shows this information for the control condition and the experimental conditions separately). Fresh and frozen meats represent 24% of the total expenditure on average, while sauces only represent 6%. Also, purchases of at least one item within food groups during the five-week study period varied from 8% for low-sodium sauces to 90% for low-sugar grains. The price per 100 g for nutritional clusters high in sugar/sodium/SAFA within the aggregate categories grains, dairy and meat are higher compared to the nutritional clusters that are low or medium in sugar/sodium/SAFA content. Regarding name- and home-brand food groups, the price per 100 g of name-brand products was higher, while the purchases of name- and home-brand products was approximately equal, resulting in higher expenditures for name-brand compared to home-brand food groups (Table 2). The triangles in Fig. 1 indicate that if the price of foods increased by one percent, purchases of targeted foods decreased by approximately 0.30% to 1.10%. Overall, 26 of 36 uncompensated own-price elasticities were inelastic (i.e., less than one) (Fig. 1; symbolized by triangles). In 6 out of the 12 nutritional clusters, the own-price elasticities of clusters high in sugar/sodium/SAFA were lower than the price elasticities found in low and medium sugar/sodium/SAFA clusters.

**Table 1:** Median price, purchases, expenditure and expenditure shares for households in the nutritional clusters (excluding zero purchases)

**Aggregate food categories**^a^	**Nutritional clusters**	**Number of shopping events (% of those included compared to overall shops)**	**Price per 100 g in NZ$**		**Purchased quantity in grams**		**Expenditure in NZ$**		**Percentage of expenditure per food category out of total expenditure**
			**Median**	**IQR**	**Median**	**IQR**	**Median**	**IQR**	
**Beverages**	Low-sugar	2386 (56%)	3.38	5.25	300	1400	7.89	8.29	9%
	Medium-sugar	3530 (83%)	0.21	0.07	3500	3000	7.02	7.46	
	High-sugar	582 (14%)	0.52	0.91	1000	1135	3.79	2.21	
**Grains**	Low-sugar	3815 (90%)	0.52	0.33	1982	2300	10.52	11.22	11%
	Medium-sugar	1361 (32%)	1.11	0.73	500	440	5.50	3.97	
	High-sugar	932 (22%)	1.14	1.28	650	1000	6.00	4.49	
	Low-sodium	2540 (60%)	0.32	0.29	1500	1650	5.38	5.82	
	Medium-sodium	3696 (87%)	0.68	0.41	1360	1470	9.23	10.48	
	High-sodium	906 (21%)	1.49	1.23	350	530	4.46	1.84	
**Dairy**	Low-sodium	3599 (85%)	0.27	0.14	3000	2375	8.75	8.62	12%
	Medium-sodium	474 (11%)	2.54	2.11	250	300	6.52	4.61	
	High-sodium	2181 (51%)	1.85	1.59	900	750	11.15	7.24	
	Low-SAFA	1271 (30%)	0.25	0.44	2000	1250	5.38	3.82	
	Medium-SAFA	3114 (73%)	0.25	0.14	2225	2000	7.04	6.65	
	High-SAFA	2455 (58%)	1.81	1.47	900	650	11.75	9.15	
**Meat**	Low-SAFA	3065 (72%)	1.91	0.71	750	760	13.14	14.38	24%
	Medium-SAFA	3155 (74%)	1.93	0.62	855	900	16.31	17.28	
	High-SAFA	2512 (59%)	1.79	0.78	770	770	12.63	12.40	
	Low-sodium	3491 (82%)	1.95	0.61	1210	1250	23.51	25.56	
	Medium-sodium	2617 (61%)	1.72	0.62	600	610	9.90	10.64	
	High-sodium	1522 (36%)	1.97	1.14	480	500	10.31	9.04	
**Sauces and seasonings**	Low-sugar	1614 (38%)	1.18	1.35	495	470	4.89	4.27	6%
	Medium-sugar	1212 (29%)	1.19	0.97	400	300	4.29	2.71	
	High-sugar	1206 (28%)	1.10	0.60	520	460	5.04	4.28	
	Low-sodium	357 (8%)	1.25	0.74	500	30	6.63	3.17	
	Medium-sodium	1679 (39%)	0.90	0.62	500	581	4.87	4.53	
	High-sodium	1664 (39%)	1.44	0.88	400	380	5.18	4.83	
**Snacks**	Low-sugar	2589 (61%)	1.64	0.83	350	390	5.47	6.21	11%
	Medium-sugar	1840 (43%)	1.33	1.26	420	750	5.52	4.90	
	High-sugar	2828 (66%)	1.77	0.84	490	690	8.40	9.42	
	Low-sodium	2675 (63%)	1.74	1.43	500	1062	7.93	9.02	
	Medium-sodium	2723 (64%)	1.57	0.66	450	540	6.99	7.92	
	High-sodium	1871 (44%)	1.66	0.75	250	270	4.19	4.32	
	Low-SAFA	2011 (47%)	1.43	1.04	375	375	4.51	4.65	
	Medium-SAFA	1729 (41%)	1.87	0.98	250	290	4.78	4.84	
	High-SAFA	3163 (74%)	1.66	0.86	600	925	9.81	11.67	

^a^ Milk is considered a beverage as well as a dairy product – all other foods and beverages are mutually exclusive within the nutritional clusters

*Abbreviations*: *IQR* Interquartile Range

**Table 2:** Median price, purchases, expenditure and expenditure shares for households in name- and home-brand food groups (excluding zero purchases)

**Aggregate food categories**	**Name- and home-brand food groups**	**Number of shops (% of shops included compared to overall shops)**	**Price per 100 g in NZ$**		**Purchased quantity in grams**		**Expenditure in NZ$**	
			**Median**	**IQR**	**Median**	**IQR**	**Median**	**IQR**
**Beverages**	Name-brand	3189 (75%)	0.43	0.91	2000	3226	10.13	12.04
	Home-brand	2447 (58%)	0.19	0.06	2000	2000	4.89	4.69
**Grains**	Name-brand	3474 (82%)	0.81	0.48	1400	1555	10.74	11.98
	Home-brand	2619 (62%)	0.30	0.16	1500	1890	4.71	5.73
**Snacks**	Name-brand	3390 (80%)	1.76	0.72	647	870	10.99	13.46
	Home-brand	2021 (48%)	1.16	0.96	390	700	5.17	5.05

*Abbreviations*: *IQR* Interquartile Range

The uncompensated cross-price elasticities show substitutive (Fig. 1; symbolized by the dots above the zero) as well as complementary (Fig. 1; symbolized by the dots below the zero) relationships with other foods within the same nutritional cluster. Larger uncertainty intervals apparent in Fig. 1 correspond to more zero-purchases within the three levels found in nutritional clusters (Table 1; column 3). Exact price elasticities displayed in Fig. 1 can be found in Supplementary Table 6. Statistically significant substitution and complementary effects were found in n = 16 (22%) and n = 26 (36%) of all cases, respectively. Of these significant cross-price elasticities, n = 11 substitutions and n = 16 complements were larger than the cut-off of 0.10. Patterns of substitution or complementary effects differed widely between nutritional clusters, i.e., no consistent pattern of substitution or complementary purchasing was evident. Within food groups with a high level of sugar/sodium/SAFA (i.e., food groups likely to be targeted by a health-related tax), some beneficial substitution and complementary effects were found in the food categories dairy, meats, and snacks. For example, a one percent increase in the price of high-sugar snacks was associated with 0.09% (SE 0.03) decrease in purchases of low-sugar snacks and a 0.20% (SE 0.03) increase in purchases of medium-sugar snacks (Supplementary Table 6).

**Fig. 2:**
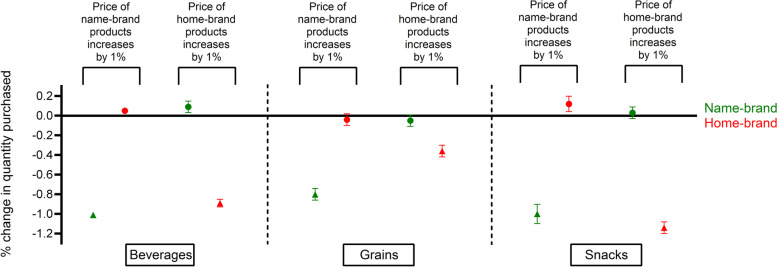
Change in demand (%) as a response to a 1% price increase in name- and home-brand categories adjusted for demographic variables. Own-price elasticities are displayed as triangles and cross-price elasticities are displayed as dots. Dots above the line represent substitution effects and dots below the line represent complementary effects

For beverages and grains, own-price elasticities of name-brand products were higher than those of home-brand products (Fig. 2 and Supplementary Table 8). Statistically significant substitution effects were found in n = 3 (50%) of cases, but only in one case was this above the 0.10 threshold; a one percent price increase in name-brand snacks was associated with a 0.12% (SE 0.04) increase in purchases of home-brand snacks.

## Discussion

We investigated the effect of health-related food taxes on consumer purchases of targeted and non-targeted alternatives within the same food group in a supermarket setting. This led to the better understanding of the potential unintended product substitution and complementary effects. As also demonstrated in previous Price ExaM Studies [10, 11], taxing products high in sugar/sodium/SAFA (i.e., unhealthier foods) leads to modest decreases in purchases of targeted products according to their own-price elasticities. Uncompensated price elasticities showed both complementary and substitution effects within some specific unhealthier food clusters. Furthermore, substitutions from name-brand to home-brand beverages and snacks were observed, but these were relatively small (i.e., most were smaller than 0.10).

We found that if the prices of foods increased by 10%, purchases of targeted foods typically decreased by approximately 3% to 11%. Most uncompensated own-price elasticities were inelastic (i.e., smaller than 1 in absolute value). This finding is in line with previous studies [9, 30] and unsurprising given the fact that food is considered a necessity. In approximately half of the clusters, the own-price elasticities of clusters high in sugar/sodium/SAFA were lower compared to clusters with low and medium amounts of sugar/sodium/SAFA. The largely inelastic own-price elasticities and the even lower own-price elasticities of clusters high in sugar/sodium/SAFA compared to clusters with lower amounts of sugar/sodium/SAFA suggests that when implementing taxes to achieve health goals, it may be preferable to apply substantive taxes (i.e., above 20% [31, 32]). Although the percentage decrease in purchases is disproportionate to the percentage increase in price, larger taxes will lead to larger purchasing changes compared to smaller taxes.

We observed that health-related taxes alter food and beverage purchases in a rather complex fashion, with only some of the substitution and complementary effects supporting the goal of the health-related taxes. Patterns in uncompensated cross-price elasticities varied between food categories, where some substitution or complementary effects towards healthier options were observed in snacks, meat and dairy and no effects were observed in beverages, grains and sauces. When it came to substitutions from name-brand foods to home-brand foods, we found that within two of the three food categories examined (i.e., beverages and snacks), cheaper and equally unhealthy home-brand foods were substituted for name-brand foods.

Given the detailed data needed to estimate these cross-price elasticities, few similar studies are available with which to compare our results. The finding that name- and home-brand products are substitutes has been reported previously, but including smaller food groups (e.g., breakfast cereals and mayonnaise) [14]. Regarding within food group substitutions to healthier alternatives, most studies to date have focused on beverages. Our findings suggest that if the price of sugary beverages increases, individuals purchase fewer taxed sugary beverages, but there is no change in the purchases of healthier beverages. However, previous evidence suggests that a SSB tax leads to substitutions with water (albeit not at a statistically significant level) [6, 33, 34]. Furthermore, similar to this study, a paper investigating cross-price elasticities within nutritionally clustered food groups using supermarket food purchasing data found relatively small within food group substitution effects [30].

The results of the uncompensated price elasticities analyses seem to imply that within food group substitutions and complements contributed minimally to the effects found in the main Price ExaM Study where a saturated fat, sugar and salt tax led to a 16%, 5% and 20% decrease in purchases of saturated fat, sugar and sodium as a percentage of total energy [10]. While the current study found limited substitution or complementary purchases, it is possible that between food group substitutions have taken place [30]. A study that investigated between food groups substitutions found for example that a 10% price increase in high-sugar soft drinks led to a 1% increase in the purchases of chocolate and confectionary [21]. Based on the small health-related substitution and complementary effects found in this study, it seems that the indirect effects of health-related food taxes do not necessarily enhance the overall health effects. However, these strategies also do not seem to lead to any unintended effects either.

While there is a wealth of evidence demonstrating that health-related taxes lead to healthier food purchases [4, 5, 9], it is still important to further investigate potential unintended effects of health-related taxes on food purchases and consumption. It is likely that not many studies have attempted to calculate cross-price elasticities within food groups due to the detailed and large dataset required. While we attempted to describe the unintended effects of health-related taxes on food purchases of close substitutes, our estimations may suffer from selection bias as the dataset is censored because zero-purchasers were excluded. This bias may differ by food groups; the percentage of shopping events included in the sauces and seasoning category ranged from 8 to 39% of the total observations, while this percentage in the meat category ranged from 36 to 82%. A previous study compared a quadratic AIDS model adjusted for zero purchases to a quadratic AIDS model unadjusted for zero purchases, and found that the price elasticities in the unadjusted model were smaller than those found in the adjusted model [12]. The results from this previous study suggest that our results provide a conservative estimate. Nevertheless, the AIDS model was preferred over more simple models that account for zero purchases (e.g., double hurdle models) as it satisfies micro-economics restrictions such as adding-up. Also, this study makes an implicit assumption that substitutions only take place *within* food groups; *between* food group substitutions could also take place. This may contradict the basis of AIDS models imposing prior constraints on the substitution process. Nevertheless, our approach implicitly assumed a multistage demand model [35] and this multistage demand model has been previously used in combination with an AIDS model [36]. More research regarding substitution effects of name- and home-brand products within other food categories is needed. Also, the effects of price changes on substitutions from unhealthy to healthier products within other categories (e.g., ready-made meals) could be investigated, but would require an even larger sample size than the present. Furthermore, as responses to price changes likely vary by cultural norms, more culture-specific and context-specific research is needed [10].

By gaining more insight into substitution and complementary effects, health-related taxes can be adapted correspondingly to further increase its effectiveness on food purchases. One example of investigating the impact of unintended cross price elasticities is to model food pricing interventions through a multi-state lifetable in order to calculate health outcomes for a specific population [11]. However, it should be noted that as our uncompensated cross-price elasticities were estimated based on the assumption that food expenditure was held constant, some adjustment must be made (e.g., using the total food expenditure elasticity) when using the price elasticities to calculate food purchases [37].

Strengths of this study included the randomized repeated measures design allowing us to collect precise and specific food price elasticity data [10] and the relatively large sample size. This allowed for the construction of nutritional clusters that represent distinct sets of products within various food categories, which is often not possible when using subjective measures and less-detailed data. A limitation of this study—not including the limitations with regards to the AIDS model described above—is that despite that the VS environment has been validated and reflects real life purchases, virtual purchases may not be directly generalizable to the real world. For example, price changes in the virtual environment were not conveyed to participants, whereas real-life price changes are often communicated to consumers, likely resulting in larger effects [38].

## Conclusion

This study examined the impact of health-related food taxes on purchasing of close substitute foods. Analyses presented suggest that food taxes lead to minimal *within* food group substitutions or complements. Given the relatively low own-price elasticities and the limited health-related substitution and complementary effects, relatively high tax rates are needed to substantively increase the proportion of healthy food purchases at the population level.

## Supplementary Information

**Additional file 1: Supplementary Table 1**. Food category and corresponding food classification to food groups based on sugar, sodium or SAFA content of products. **Supplementary File 2a**. List of smaller food groups found within the aggregate food categories. **Supplementary File 2b**. List of smaller food groups found within the aggregate home-brand and name-brand categories. **Supplementary Table 3**. Median nutritional content per serving and percentage of healthy foods within the aggregated categories. **Supplementary Table 4a**. Percentage change in price, expenditure and quantity between the control condition and the experimental conditions for nutrient-based food groups. **Supplementary Table 5**. Mean expenditure elasticities and standard errors for all nutrient-based food groups. **Supplementary Table 6**. Uncompensated elasticities and corresponding standard errors (shaded boxes showing own price elasticities, others being cross price elasticities). **Supplementary Table 7**. Compensated elasticities and corresponding standard errors (shaded boxes showing own price elasticities, others being cross price elasticities). **Supplementary Table 8**. Price elasticities and standard errors from the double log model for name- and home-brand products within the food categories.

## Data Availability

Requests for de-identified individual participant data or study documents will be considered where the proposed use aligns with public positive purposes, does not conflict with other requests or planned use by the trial steering committee, and the requestor is willing to sign a data access agreement. Contact is through NN. The protocol for the Price ExaM Study is available from WEW on request, and the protocol for the current study is available from JCH on request.
