# Supplementary material for: Impact of taxes on purchases of close substitute foods: analysis of cross-price elasticities using data from a randomized experiment
Source: Nutr J. 2021 Sep 7;20:75. doi: 10.1186/s12937-021-00736-y (PMC8424883; doi:10.1186/s12937-021-00736-y)
Supplement: Supplementary file 1 — Additional file 1: Supplementary Table 1. Food category and corresponding food classification to food groups based on sugar, sodium or SAFA content of products. Supplementary File 2a. List of smaller food groups found within the aggregate food categories. Supplementary File 2b. List of smaller food groups found within the aggregate home-brand and name-brand categories. Supplementary Table 3. Median nutritional content per serving and percentage of healthy foods within the aggregated categories. Supplementary Table 4a. Percentage change in price, expenditure and quantity between the control condition and the experimental conditions for nutrient-based food groups. Supplementary Table 5. Mean expenditure elasticities and standard errors for all nutrient-based food groups. Supplementary Table 6. Uncompensated elasticities and corresponding standard errors (shaded boxes showing own price elasticities, others being cross price elasticities). Supplementary Table 7. Compensated elasticities and corresponding standard errors (shaded boxes showing own price elasticities, others being cross price elasticities). Supplementary Table 8. Price elasticities and standard errors from the double log model for name- and home-brand products within the food categories. [file 12937_2021_736_MOESM1_ESM.docx]

**SUPPLEMENTARY FILES**

Supplementary Table 1. Food category and corresponding food classification to food groups based on sugar, sodium or SAFA content of products

| **Food category** | **Low cut-off value** | **Middle cut-off value** | | **High cut-off value** | | **Nutritional clusters** |
| --- | --- | --- | --- | --- | --- | --- |
| **Beverages** | <2.5g sugar per 100ml | 2.5-11.25g sugar per 100ml | | >11.25g sugar per 100ml | | Low-sugar beverages |
|  |  |  |  |  |  | Medium-sugar beverages |
|  |  |  |  |  |  | High-sugar beverages |
| **Grain products (cereals and bread)** | <5g sugar per 100g | 5-22.5g sugar per 100g | | >22.5g sugar per 100g | | Low-sugar grains |
|  |  |  |  |  |  | Medium-sugar grains |
|  |  |  |  |  |  | High-sugar grains |
|  | <120mg sodium per 100g | 120-600mg sodium per 100g | | >600mg sodium per 100g | | Low-sodium grains |
|  |  |  |  |  |  | Medium-sodium grains |
|  |  |  |  |  |  | High-sodium grains |
| **Dairy** | For food: <120mg sodium per 100g  For milk:  <120mg sodium 100ml | For food:  120-600mg sodium per 100g  For milk:  120-300mg sodium per 100ml | | For food:  >600mg sodium per 100g  For milk:  >300mg sodium per 100ml | | Low-sodium dairy |
|  |  |  |  |  |  | Medium-sodium dairy |
|  |  |  |  |  |  | High-sodium dairy |
|  | For food:  <1.5g SAFA per 100g  For milk:  <0.75g SAFA per 100ml | For food:  1.5-5g SAFA per 100g  For milk:  0.75-2.5g SAFA per 100ml | | For food:  >5g SAFA per 100g  For milk:  >2.5g SAFA per 100ml | | Low-SAFA dairy |
|  |  |  |  |  |  | Medium-SAFA dairy |
|  |  |  |  |  |  | High-SAFA dairy |
| **Fresh and frozen meat** | <1.5g SAFA per 100g | 1.5-5g SAFA per 100g | | >5g SAFA per 100g | | Low-SAFA meats |
|  |  |  |  |  |  | Medium-SAFA meats |
|  |  |  |  |  |  | High-SAFA meats |
|  | <120mg sodium per 100g | 120-600mg sodium per 100g | | >600mg sodium per 100g | | Low-sodium meats |
|  |  |  |  |  |  | Medium-sodium meats |
|  |  |  |  |  |  | High-sodium meats |
| **Sauces and seasonings** | <120mg sodium per 100g | 120-600mg sodium per 100g | | >600mg sodium per 100g | | Low-sodium sauces |
|  |  |  |  |  |  | Medium-sodium sauces |
|  |  |  |  |  |  | High-sodium sauces |
|  | <5g sugar per 100g | | 5-22.5g sugar per 100g | | >22.5g sugar per 100g | Low-sugar sauces |
|  |  |  |  |  |  | Medium-sugar sauces |
|  |  |  |  |  |  | High-sugar sauces |
| **Snack foods**  **and desserts** | <120mg sodium per 100g | 120-600mg sodium per 100g | | >600mg sodium per 100g | | Low-sodium snacks |
|  |  |  |  |  |  | Medium-sodium snacks |
|  |  |  |  |  |  | High-sodium snacks |
|  | <1.5g SAFA per 100g | 1.5-5g SAFA per 100g | | >5g SAFA per 100g | | Low-SAFA snacks |
|  |  |  |  |  |  | Medium-SAFA snacks |
|  |  |  |  |  |  | High-SAFA snacks |
|  | <5g sugar per 100g | 5-22.5g sugar per 100g | | >22.5g sugar per 100g | | Low-sugar snacks |
|  |  |  |  |  |  | Medium-sugar snacks |
|  |  |  |  |  |  | High-sugar snacks |

Supplementary File 2a. List of smaller food groups found within the aggregate food categories

| **Food category** | **Nutrient-based food groups** | **Smaller food groups found within nutritional clusters** |
| --- | --- | --- |
| **Beverages** | Low-sugar beverages | *Tea* |
|  |  | *Coffee* |
|  |  | *Sugar free soft and energy drinks* |
|  |  | *Flavored water* |
|  |  | *Water* |
|  |  | *Soy milk* |
|  | Medium-sugar beverages | *Dairy milk* |
|  |  | Fruit and vegetable juices |
|  |  | Sugar-sweetened soft drinks |
|  |  | Flavored milk |
|  | High-sugar beverages | Fruit and vegetable juices |
|  |  | Sugar-sweetened soft drinks |
|  |  | *Flavored soy milk* |
|  |  | Flavored milk |
| **Grain products (cereals and bread)** | Low-sugar grains | *Unflavored rice (white and wholegrain rice)* |
|  |  | *Flavored white rice* |
|  |  | *All pastas (i.e., packet, canned, plain dry and fresh)* |
|  |  | *Flour* |
|  |  | *Noodles* |
|  |  | Oats |
|  |  | *All breads (i.e., mixed grain, white and other)* |
|  |  | *Couscous* |
|  |  | *Wheat breakfast cereals* |
|  | Medium-sugar grains | *Muesli* |
|  |  | *Bagels* |
|  |  | *Savory pastries* |
|  |  | *Quinoa* |
|  |  | Breakfast cereals |
|  | High-sugar grains | *Oats* |
|  |  | *Sweet pastries* |
|  |  | Breakfast cereals |
|  |  | *Sugar* |
|  | Low-sodium grains | *Sweet pastries* |
|  |  | *Wholegrain rice* |
|  |  | Flour |
|  |  | *Muesli* |
|  |  | Noodles |
|  |  | *Oats* |
|  |  | *Couscous* |
|  |  | *Plain dry pasta* |
|  |  | *Quinoa* |
|  |  | *Breakfast cereal* |
|  |  | *Sugar* |
|  |  | *Unflavored rice (white and wholegrain rice)* |
|  | Medium-sodium grains | *Mixed grain sandwich bread* |
|  |  | Noodles |
|  |  | Other bread |
|  |  | Packet pasta |
|  |  | *Sweet pastries* |
|  | High-sodium grains | Flour |
|  |  | Noodles |
|  |  | Other bread |
|  |  | Packet pasta |
|  |  | *Savory pastries* |
|  |  | *White sandwich bread* |
|  |  | *Flavored white rice* |
| **Dairy** | Low-sodium dairy | *Sour crème & crème fraiche* |
|  |  | *Flavored yoghurt* |
|  |  | *Light/low fat yoghurt* |
|  |  | *Cream* |
|  |  | *Skimmed milk* |
|  |  | Flavored milk |
|  |  | *Dairy milk* |
|  | Medium-sodium dairy | *Coconut milk* |
|  |  | Flavored milk |
|  |  | *Fresh cheese (i.e., mozzarella, cottage and ricotta)* |
|  |  | Softer cheese (i.e., brie) |
|  | High-sodium dairy | *Hard cheese (i.e., 30+ and 48+)* |
|  |  | *Blue cheese* |
|  |  | Softer cheese (i.e., camembert) |
|  |  | *Processed cheese (e.g., spray can cheese)* |
|  | Low-SAFA dairy | Flavored yoghurt |
|  |  | *Skimmed milk* |
|  |  | Light/low fat yoghurt |
|  |  | Fresh cheese (i.e., cottage cheese) |
|  | Medium-SAFA dairy | *Dairy milk* |
|  |  | *Flavored milk* |
|  |  | Flavored yoghurt |
|  |  | Light/low fat yoghurt |
|  |  | Fresh cheese (i.e., cottage cheese) |
|  | High-SAFA dairy | *Hard cheese (i.e., 30+ and 48+)* |
|  |  | *Blue cheese* |
|  |  | *Softer cheese (i.e., camembert)* |
|  |  | *Processed cheese (e.g., spray can cheese)* |
|  |  | *Cream* |
|  |  | *Coconut milk* |
|  |  | Fresh cheese (i.e., mozzarella and ricotta) |
| **Fresh and frozen meat** | Low-SAFA meats | Fatty fish (i.e., tuna) |
|  |  | *Unflavored lean fish* |
|  |  | *Processed lean fish* |
|  |  | Poultry |
|  |  | Processed meat |
|  |  | *Tofu* |
|  |  | *Falafel* |
|  | Medium-SAFA meats | Fatty fish (i.e., salmon, tuna and sardines) |
|  |  | Poultry |
|  |  | Processed fish |
|  |  | Processed meat |
|  |  | Red meat |
|  |  | *Vegetarian burgers* |
|  | High-SAFA meats | Fatty fish (i.e., salmon) |
|  |  | *Processed poultry (i.e., sausages)* |
|  |  | Processed meat |
|  |  | Red meat |
|  | Low-sodium meats | Fatty fish (i.e., salmon) |
|  |  | *Unflavored lean fish* |
|  |  | *Poultry* |
|  |  | Red meat |
|  |  | *Tofu* |
|  | Medium-sodium meats | Fatty fish (i.e., tuna and sardines) |
|  |  | *Processed lean fish* |
|  |  | Processed poultry |
|  |  | Processed meat |
|  |  | Red meat |
|  |  | *Falafel* |
|  | High-sodium meats | Fatty fish (i.e., salmon and anchovies) |
|  |  | Processed poultry |
|  |  | Processed meat |
|  |  | *Vegetarian burgers* |
|  |  | Red meat |
| **Sauces and seasonings** | Low-sodium sauces | *Sweet sauces* |
|  | Medium-sodium sauces | Asian sauces |
|  |  | *Chutney* |
|  |  | Dips |
|  |  | Gravies |
|  |  | Meal-based sauces |
|  |  | Pasta sauce |
|  |  | *Pickles and vinegar* |
|  |  | Wok sauce |
|  | High-sodium sauces | Asian sauces |
|  |  | *Chili sauce* |
|  |  | Dips |
|  |  | Gravies |
|  |  | *Stock* |
|  |  | *Hummus* |
|  |  | *Mayonnaise* |
|  |  | *Marinade* |
|  |  | Meal-based sauces |
|  |  | *Meat accompaniment* |
|  |  | *Mustard* |
|  |  | Pasta sauce |
|  |  | *Paste* |
|  |  | *Salad dressing* |
|  |  | *Seasoning* |
|  |  | *Soy sauce* |
|  |  | Wok sauce |
|  | Low-sugar sauces | Asian sauces |
|  |  | Dips |
|  |  | *Gravies* |
|  |  | *Stock* |
|  |  | *Hot sauce* |
|  |  | *Hummus* |
|  |  | Meal-based sauces |
|  |  | Pasta sauce |
|  |  | Paste |
|  |  | *Soy sauce* |
|  |  | Wok sauce |
|  | Medium-sugar sauces | Dips |
|  |  | Meal-based sauces |
|  |  | Meat accompaniment |
|  |  | *Mustard* |
|  |  | Pasta sauce |
|  |  | Paste |
|  |  | Pickles and vinegar |
|  |  | Salad dressing |
|  |  | Wok sauce |
|  | High-sugar sauces | Asian sauces |
|  |  | Meat accompaniment |
|  |  | Pasta sauce |
|  |  | Paste |
|  |  | Pickles and vinegar |
|  |  | Salad dressing |
|  |  | Wok sauce |
|  |  | *Sweet sauces* |
|  |  | *Chili sauce* |
|  |  | *Chutney* |
|  |  | *Mayonnaise* |
| **Snack foods**  **and desserts** | Low-sodium snacks | *Confectionary* |
|  |  | Cereal-based bars |
|  |  | *Chocolate-based confectionary* |
|  |  | *Fruit bars* |
|  |  | *Gum* |
|  |  | *Ice cream* |
|  |  | *Nut-based bars* |
|  |  | *Milk-based desserts* |
|  |  | Nuts |
|  | Medium-sodium snacks | *Pie and cake* |
|  |  | Cereal-based bars |
|  |  | Chips |
|  |  | Chocolate-based confectionary |
|  |  | *Muffins* |
|  |  | Nuts |
|  |  | Plain dry biscuits |
|  |  | *Pudding* |
|  |  | *Savory biscuits* |
|  |  | *Sweet filled biscuits* |
|  |  | *Sweet unfilled biscuits* |
|  |  | *Warm snacks* |
|  | High-sodium snacks | *Chips* |
|  |  | *Popcorn* |
|  |  | Nuts |
|  |  | *Other savory snacks* |
|  |  | Plain dry biscuits |
|  | Low-SAFA snacks | Pie and cake |
|  |  | Confectionary |
|  |  | Fruit bars |
|  |  | *Gum* |
|  |  | *Fruit-based desserts* |
|  |  | Muffins |
|  |  | *Other savory snacks* |
|  |  | Plain dry biscuits |
|  |  | Savory biscuits |
|  |  | *Warm snacks* |
|  | Medium-SAFA snacks | Pie and cake |
|  |  | Cereal-based bars |
|  |  | Chocolate-based confectionary |
|  |  | Fruit bars |
|  |  | Chips |
|  |  | Muffins |
|  |  | Nut-based bars |
|  |  | Nuts |
|  |  | *Pudding* |
|  |  | Plain dry biscuits |
|  |  | Savory biscuits |
|  | High-SAFA snacks | Pie and cake |
|  |  | Confectionary |
|  |  | Cereal-based bars |
|  |  | Chips |
|  |  | Chocolate-based confectionary |
|  |  | *Milk-based desserts* |
|  |  | Nut-based bars |
|  |  | Nuts |
|  |  | Savory biscuits |
|  |  | *Sweet filled biscuits* |
|  |  | *Sweet unfilled biscuits* |
|  | Low-sugar snacks | Chips |
|  |  | *Gum* |
|  |  | Muffins |
|  |  | *Popcorn* |
|  |  | Savory biscuits |
|  |  | *Warm snacks* |
|  |  | Nuts |
|  |  | Other savory snacks |
|  |  | *Plain dry biscuits* |
|  | Medium-sugar snacks | Chips |
|  |  | Fruit-based desserts |
|  |  | Milk-based desserts |
|  |  | Nut-based bars |
|  |  | *Other savory snacks* |
|  |  | Pie and cake |
|  |  | Savory biscuits |
|  |  | Sweet unfilled biscuits |
|  | High-sugar snacks | Pie and cake |
|  |  | *Confectionary* |
|  |  | *Chocolate-based confectionary* |
|  |  | *Cereal-based bars* |
|  |  | Nut-based bars |
|  |  | Nuts |
|  |  | Sweet unfilled biscuits |
|  |  | *Toppings* |
|  |  | *Pudding* |
|  |  | *Sweet filled biscuits* |
|  |  | *Fruit bars* |
|  |  | Fruit-based desserts |
|  |  | Milk-based desserts |
|  |  | Muffins |

Products in italics are exclusively found in one nutrient-based food group

Supplementary File 2b. List of smaller food groups found within the aggregate home-brand and name-brand categories

| **Food category** | **Aggregate categories** | **Smaller food groups found within nutrient-based food groups** |
| --- | --- | --- |
| **Beverages** | Name-brand beverages | Coffee |
|  |  | Tea |
|  |  | Fruit and vegetable juices |
|  |  | Flavored water |
|  |  | Dairy milk |
|  |  | *Soy milk* |
|  |  | *Water* |
|  |  | Trimmed milk |
|  |  | *Flavored milk* |
|  |  | Soft drinks |
|  |  | *Sugar-free soft drinks* |
|  |  | *Energy drinks* |
|  | Home-brand beverages | Fruit and vegetable juices |
|  |  | Tea |
|  |  | Flavored water |
|  |  | Dairy milk |
|  |  | Trimmed milk |
|  |  | Soft drinks |
| **Grain products (cereals and bread)** | Name-brand grains | *Unflavored rice (white and wholegrain rice)* |
|  |  | *Flavored white rice* |
|  |  | All pastas (i.e., packet, canned, plain dry and fresh) |
|  |  | *Flour* |
|  |  | *Noodles* |
|  |  | *Oats* |
|  |  | All breads (i.e., mixed grain, white and other) |
|  |  | *Couscous* |
|  |  | *Wheat breakfast cereals* |
|  |  | Breakfast cereals |
|  |  | *Muesli* |
|  |  | *Quinoa* |
|  |  | Sugar |
|  | Home-brand grains | Other bread |
|  |  | White bread |
|  |  | Fresh pasta |
|  |  | Sugar |
|  |  | Breakfast cereals |
| **Snacks** | Name-brand snacks | Cake |
|  |  | Confectionary |
|  |  | Cereal-based bars |
|  |  | Chips |
|  |  | Milk-based deserts |
|  |  | Muffins |
|  |  | Nuts |
|  |  | Plain dry biscuits |
|  |  | Savory biscuits |
|  |  | Sweet unfilled biscuits |
|  |  | Toppings |
|  |  | *Chocolate-based confectionary* |
|  |  | *Fruit bars* |
|  |  | *Gum* |
|  |  | *Fruit-based deserts* |
|  |  | *Nut-based bars* |
|  |  | *Other savory snacks* |
|  |  | *Popcorn* |
|  |  | *Pudding* |
|  |  | *Sweet filled biscuits* |
|  |  | *Warm snacks* |
|  | Home-brand snacks | Cake |
|  |  | Confectionary |
|  |  | Cereal-based bars |
|  |  | Chips |
|  |  | Milk-based deserts |
|  |  | Muffins |
|  |  | Nuts |
|  |  | Plain dry biscuits |
|  |  | Savory biscuits |
|  |  | Sweet unfilled biscuits |
|  |  | Toppings |

Products in italics are only found in the name-brand food groups

Supplementary Table 3. Median nutritional content per serving and percentage of healthy foods within the aggregated categories

| **Food category** | **Nutritional clusters** | **Kilo calories** | **SAFA (g)** | **Sugar**  **(g)** | **Sodium (mg)** | **% of healthy products^1^** |
| --- | --- | --- | --- | --- | --- | --- |
| **Beverages** | Low-sugar | 1.2 | 0.0 | 0.0 | 1.3 | 94.6% |
|  | Medium-sugar | 44.2 | 0.0 | 8.9 | 11.0 | 60.4% |
|  | High-sugar | 61.0 | 0.8 | 14.0 | 6.7 | 50.0% |
|  | Name-brand | 37.3 | 0.0 | 1.0 | 5.0 | 78.4% |
|  | Home-brand | 34.7 | 0.0 | 7.0 | 8.0 | 72.0% |
| **Grains** | Low-sugar | 251.0 | 0.5 | 2.3 | 422.0 | 73.6% |
|  | Medium-sugar | 365.7 | 1.5 | 8.4 | 385.5 | 46.9% |
|  | High-sugar | 385.9 | 0.8 | 30.9 | 104.0 | 31.8% |
|  | Low-sodium | 361.1 | 0.5 | 2.8 | 23.5 | 82.6% |
|  | Medium-sodium | 260.5 | 0.5 | 3.1 | 407.5 | 68.1% |
|  | High-sodium | 340.6 | 2.1 | 4.3 | 900.0 | 4.5% |
|  | Name-brand | 319.6 | 0.7 | 3.3 | 380.0 | 59.7% |
|  | Home-brand | 310.7 | 0.5 | 3.2 | 190.0 | 80.0% |
| **Dairy** | Low-sodium | 89.7 | 1.9 | 4.95 | 40.0 | 66.7% |
|  | Medium-sodium | 334.6 | 14.9 | 1.0 | 430.0 | 50.0% |
|  | High-sodium | 358.5 | 19.2 | 1.0 | 708.0 | 16.2% |
|  | Low-SAFA | 47.8 | 0.4 | 4.8 | 41.0 | 100.0% |
|  | Medium-SAFA | 91.0 | 1.9 | 10.0 | 41.0 | 68.2% |
|  | High-SAFA | 358.5 | 19.1 | 1.0 | 687.0 | 19.2% |
| **Fresh and frozen meat** | Low-SAFA | 113.3 | 0.8 | 1.0 | 310.0 | 81.4% |
|  | Medium-SAFA | 181.2 | 2.4 | 1.0 | 370.0 | 57.1% |
|  | High-SAFA | 248.6 | 6.8 | 1.0 | 559.5 | 2.9% |
|  | Low-sodium | 181.8 | 1.7 | 1.0 | 61.0 | 84.0% |
|  | Medium-sodium | 197.0 | 1.8 | 1.2 | 355.0 | 61.5% |
|  | High-sodium | 226.1 | 4.4 | 1.1 | 890.0 | 8.6% |
| **Sauces and seasonings** | Low-sugar | 94.8 | 1.0 | 2.8 | 459.0 | 28.3% |
|  | Medium-sugar | 154.7 | 1.0 | 10.2 | 614.0 | 11.9% |
|  | High-sugar | 163.7 | 0.1 | 31.1 | 530.0 | 0.0% |
|  | Low-sodium | 285.6 | 0.0 | 67.8 | 13.0 | 7.4% |
|  | Medium-sodium | 95.1 | 0.5 | 4.4 | 380.0 | 29.9% |
|  | High-sodium | 175.7 | 1.0 | 10.6 | 1369.5 | 1.6% |
| **Snack foods and deserts** | Low-sugar | 435.0 | 2.25 | 1.8 | 525.0 | 26.5% |
|  | Medium-sugar | 401.5 | 5.4 | 14.1 | 280.0 | 23.1% |
|  | High-sugar | 439.8 | 8.8 | 41.0 | 127.0 | 2.7% |
|  | Low-sodium | 395.55 | 5.8 | 30.9 | 37.0 | 17.2% |
|  | Medium-sodium | 437.4 | 8.1 | 28.2 | 300.0 | 9.5% |
|  | High-sodium | 466.1 | 2.7 | 3.4 | 780.0 | 6.7% |
|  | Low-SAFA | 319.6 | 0.5 | 13.4 | 200.0 | 29.9% |
|  | Medium-SAFA | 411.1 | 3.15 | 19.2 | 265.0 | 23.5% |
|  | High-SAFA | 475.6 | 11.1 | 30.8 | 175.0 | 1.6% |
|  | Name-brand | 437.4 | 6.5 | 27.1 | 180.0 | 12.3% |
|  | Home-brand | 420.7 | 5.9 | 24.0 | 250.0 | 11.1% |

^1^ All fresh fruit and vegetables, fresh fish, and packaged foods eligible to carry a health claim based on the Nutrient Profiling Scoring Criterion were classified as healthy

Supplementary Table 4a. Percentage change in price, expenditure and quantity between the control condition and the experimental conditions for nutrient-based food groups

| Food categories | Nutritional clusters | N control condition | N experim-ental conditions^1^ | Price per 100g in NZ$ in control condition | | Price per 100g in NZ$ in experimental conditions^1^ | |  | Expenditure in NZ$ in control condition | | Expenditure in NZ$ in experimental conditons^1^ | |  | Purchased quantity in grams in control condition | | Purchased quantity in grams in experimental conditions^1^ | |  |
| --- | --- | --- | --- | --- | --- | --- | --- | --- | --- | --- | --- | --- | --- | --- | --- | --- | --- | --- |
|  |  |  |  | Med | IQR | Med | IQR | % change^2^ | Med | IQR | Med | IQR | % change^2^ | Med | IQR | Med | IQR | % change^2^ |
| Beverages | Low-sugar | 377 | 2009 | 3.32 | 5.27 | 3.39 | 5.22 | 2% | 7.59 | 8.41 | 7.94 | 8.26 | 5% | 300 | 1400 | 300 | 1400 | 0% |
|  | Medium-sugar | 521 | 3009 | 0.20 | 0.05 | 0.22 | 0.08 | 10% | 6.74 | 7.33 | 7.06 | 7.49 | 5% | 3500 | 3500 | 3500 | 3000 | 0% |
|  | High-sugar | 81 | 501 | 0.48 | 0.87 | 0.55 | 0.91 | 15% | 3.63 | 2.37 | 3.79 | 2.16 | 4% | 1000 | 1275 | 1000 | 1135 | 0% |
| Grains | Low-sugar | 572 | 3243 | 0.49 | 0.26 | 0.53 | 0.34 | 8% | 9.81 | 10.58 | 10.63 | 11.30 | 8% | 2100 | 2230 | 1950 | 2340 | -7% |
|  | Medium-sugar | 192 | 1169 | 1.06 | 0.77 | 1.11 | 0.74 | 5% | 5.11 | 3.67 | 5.62 | 4.04 | 10% | 460 | 455 | 500 | 440 | 9% |
|  | High-sugar | 123 | 809 | 0.68 | 1.26 | 1.16 | 1.28 | 71% | 5.55 | 5.14 | 6.05 | 4.27 | 9% | 1000 | 1050 | 650 | 958 | -35% |
|  | Low-sodium | 375 | 2165 | 0.30 | 0.24 | 0.33 | 0.29 | 10% | 4.82 | 5.36 | 5.45 | 5.90 | 13% | 1500 | 1600 | 1500 | 1650 | 0% |
|  | Medium-sodium | 554 | 3142 | 0.62 | 0.41 | 0.69 | 0.41 | 11% | 8.67 | 9.60 | 9.34 | 10.64 | 8% | 1345 | 1480 | 1360 | 1470 | 1% |
|  | High-sodium | 151 | 755 | 1.44 | 1.22 | 1.50 | 1.21 | 4% | 4.16 | 1.62 | 4.52 | 1.90 | 9% | 350 | 550 | 350 | 530 | 0% |
| Dairy | Low-sodium | 534 | 3065 | 0.26 | 0.13 | 0.27 | 0.14 | 4% | 8.38 | 7.81 | 8.83 | 8.73 | 5% | 3000 | 2250 | 3000 | 2400 | 0% |
|  | Medium-sodium | 65 | 409 | 2.81 | 2.23 | 2.53 | 2.08 | -10% | 5.78 | 4.67 | 6.55 | 4.53 | 13% | 250 | 200 | 250 | 300 | 0% |
|  | High-sodium | 348 | 1833 | 1.54 | 1.34 | 1.91 | 1.59 | 24% | 10.55 | 6.10 | 11.32 | 7.50 | 7% | 928 | 672 | 900 | 750 | -3% |
|  | Low-SAFA | 161 | 1110 | 0.22 | 0.42 | 0.26 | 0.44 | 18% | 5.24 | 3.90 | 5.41 | 3.81 | 3% | 2000 | 1250 | 2000 | 1125 | 0% |
|  | Medium-SAFA | 472 | 2642 | 0.23 | 0.14 | 0.25 | 0.14 | 9% | 7.24 | 6.50 | 6.99 | 6.67 | -3% | 2400 | 2000 | 2225 | 2000 | -7% |
|  | High-SAFA | 392 | 2063 | 1.48 | 1.22 | 1.86 | 1.49 | 26% | 10.72 | 7.72 | 11.97 | 9.38 | 12% | 900 | 717 | 900 | 650 | 0% |
| Meat | Low-SAFA | 470 | 2595 | 1.91 | 0.71 | 1.91 | 0.72 | 0% | 13.98 | 14.41 | 13.05 | 14.35 | -7% | 800 | 770 | 740 | 745 | -8% |
|  | Medium-SAFA | 468 | 2687 | 1.88 | 0.68 | 1.94 | 0.60 | 3% | 15.42 | 16.52 | 16.59 | 17.39 | 8% | 800 | 795 | 880 | 900 | 10% |
|  | High-SAFA | 377 | 2135 | 1.75 | 0.81 | 1.80 | 0.77 | 3% | 11.79 | 11.57 | 12.73 | 12.45 | 8% | 770 | 793 | 770 | 770 | 0% |
|  | Low-sodium | 535 | 2956 | 1.93 | 0.64 | 1.95 | 0.61 | 1% | 22.36 | 24.72 | 23.65 | 25.78 | 6% | 1170 | 1260 | 1230 | 1250 | 5% |
|  | Medium-sodium | 387 | 2230 | 1.69 | 0.61 | 1.72 | 0.62 | 2% | 10.04 | 10.85 | 9.88 | 10.62 | -2% | 590 | 645 | 600 | 610 | 2% |
|  | High-sodium | 222 | 1300 | 1.89 | 1.12 | 1.97 | 1.13 | 4% | 9.72 | 8.55 | 10.37 | 9.10 | 7% | 500 | 500 | 480 | 500 | -4% |
| Sauces | Low-sugar | 242 | 1372 | 1.12 | 1.36 | 1.18 | 1.34 | 5% | 4.49 | 3.59 | 5.02 | 4.33 | 12% | 495 | 403 | 495 | 452 | 0% |
|  | Medium-sugar | 184 | 1028 | 1.18 | 0.88 | 1.19 | 1.00 | 1% | 4.20 | 2.41 | 4.31 | 2.76 | 3% | 400 | 250 | 400 | 300 | 0% |
|  | High-sugar | 179 | 1027 | 1.03 | 0.58 | 1.11 | 0.60 | 8% | 4.66 | 4.06 | 5.10 | 4.31 | 9% | 530 | 345 | 520 | 460 | -2% |
|  | Low-sodium | 59 | 298 | 1.21 | 0.79 | 1.27 | 0.71 | 5% | 6.54 | 3.80 | 6.64 | 3.09 | 2% | 500 | 30 | 500 | 30 | 0% |
|  | Medium-sodium | 237 | 1442 | 0.86 | 0.70 | 0.90 | 0.60 | 5% | 4.72 | 4.34 | 4.93 | 4.58 | 4% | 500 | 620 | 500 | 590 | 0% |
|  | High-sodium | 242 | 1422 | 1.38 | 0.73 | 1.45 | 0.91 | 5% | 4.98 | 4.62 | 5.23 | 4.85 | 5% | 405 | 380 | 400 | 380 | -1% |
| Snacks | Low-sugar | 387 | 2202 | 1.57 | 0.75 | 1.66 | 0.83 | 6% | 5.73 | 5.90 | 5.46 | 6.29 | -5% | 375 | 400 | 350 | 390 | -7% |
|  | Medium-sugar | 279 | 1561 | 1.26 | 1.38 | 1.35 | 1.24 | 7% | 5.54 | 5.35 | 5.50 | 4.83 | -1% | 420 | 900 | 418 | 730 | -1% |
|  | High-sugar | 431 | 2397 | 1.69 | 0.70 | 1.78 | 0.83 | 5% | 7.77 | 8.83 | 8.50 | 9.51 | 9% | 480 | 680 | 492 | 686 | 3% |
|  | Low-sodium | 401 | 2274 | 1.71 | 1.46 | 1.75 | 1.43 | 2% | 7.62 | 8.61 | 7.98 | 9.02 | 5% | 520 | 1386 | 500 | 1004 | -4% |
|  | Medium-sodium | 409 | 2314 | 1.45 | 0.61 | 1.60 | 0.66 | 10% | 7.07 | 8.06 | 6.95 | 7.88 | -2% | 460 | 574 | 450 | 530 | -2% |
|  | High-sodium | 286 | 1585 | 1.59 | 0.67 | 1.67 | 0.75 | 5% | 4.16 | 4.42 | 4.20 | 4.27 | 1% | 250 | 255 | 250 | 270 | 0% |
|  | Low-SAFA | 296 | 1715 | 1.41 | 0.96 | 1.43 | 1.04 | 1% | 4.60 | 4.81 | 4.50 | 4.66 | -2% | 375 | 380 | 375 | 375 | 0% |
|  | Medium-SAFA | 265 | 1464 | 1.78 | 0.92 | 1.88 | 0.98 | 6% | 5.36 | 4.99 | 4.70 | 4.82 | -12% | 293 | 375 | 250 | 278 | -15% |
|  | High-SAFA | 470 | 2693 | 1.53 | 0.82 | 1.68 | 0.86 | 10% | 9.00 | 11.43 | 9.98 | 11.66 | 11% | 600 | 1000 | 600 | 910 | 0% |

^1^ Experimental conditions include all conditions with a sugar, salt and/or SAFA tax or a subsidy on fruit and vegetables

^2^ Percentage change between the control condition and the experimental conditions

Med; Median, IQR; Interquartile range

Supplementary Table 4b. Percentage change in price, expenditure and quantity between the control condition and the experimental conditions for name- and home-brand products

| Food categories | Food groups | N in control condition | N in experim-ental conditions^1^ | Price in NZ$ in control condition | | Price in NZ$ in experimental conditions^1^ | |  | Expenditure in NZ$ in control condition | | Expenditure in NZ$ in experimental conditons^1^ | |  | Purchased quantity in grams in control condition | | Purchased quantity in grams in experimental conditions^1^ | |  |
| --- | --- | --- | --- | --- | --- | --- | --- | --- | --- | --- | --- | --- | --- | --- | --- | --- | --- | --- |
|  |  |  |  | Med | IQR | Med | IQR | % change^2^ | Med | IQR | Med | IQR | % change^2^ | Med | IQR | Med | IQR | % change^2^ |
| Beverages | Name-brand | 485 | 2704 | 0.44 | 1.14 | 0.20 | 0.06 | -55% | 9.55 | 10.93 | 10.24 | 12.24 | 7% | 2000 | 3500 | 2000 | 3000 | 0% |
|  | Home-brand | 378 | 2069 | 0.18 | 0.05 | 0.43 | 0.88 | 139% | 4.41 | 4.32 | 5.01 | 4.73 | 14% | 2000 | 2000 | 2000 | 2000 | 0% |
| Grains | Name-brand | 513 | 2961 | 0.74 | 0.46 | 0.82 | 0.50 | 11% | 10.32 | 10.89 | 10.87 | 12.16 | 5% | 1400 | 1495 | 1415 | 1700 | 1% |
|  | Home-brand | 394 | 2225 | 0.27 | 0.13 | 0.30 | 0.18 | 11% | 4.58 | 4.95 | 4.77 | 5.78 | 4% | 1500 | 1850 | 1400 | 2040 | -7% |
| Snacks | Name-brand | 511 | 2879 | 1.70 | 0.69 | 1.76 | 0.73 | 4% | 10.48 | 12.88 | 11.10 | 13.53 | 6% | 650 | 976 | 600 | 760 | -8% |
|  | Home-brand | 313 | 1708 | 1.06 | 0.97 | 1.18 | 0.95 | 11% | 4.91 | 4.89 | 5.21 | 5.09 | 6% | 390 | 650 | 375 | 500 | -4% |

^1^ Experimental conditions include all conditions with a sugar, salt and/or SAFA tax or a subsidy on fruit and vegetables

^2^ Percentage change between the control condition and the experimental conditions

Med; Median, IQR; Interquartile range

Supplementary Table 5. Mean expenditure elasticities and standard errors for all nutrient-based food groups

| **Food categories** | **Relevant nutrient** | Low levels of sugar/sodium/SAFA | | Medium levels of sugar/sodium/SAFA | | High levels of sugar/sodium/SAFA | |
| --- | --- | --- | --- | --- | --- | --- | --- |
|  |  | Elasticity | SE | Elasticity | SE | Elasticity | SE |
| Beverages | Sugar | 1.26 | 0.06 | 0.90 | 0.03 | 0.50 | 0.28 |
| Grains | Sugar | 1.10 | 0.02 | 0.63 | 0.10 | 0.51 | 0.15 |
|  | Sodium | 1.00 | 0.04 | 1.10 | 0.02 | -0.03 | 0.13 |
| Dairy | Sodium | 0.95 | 0.04 | -0.60 | 0.29 | 1.32 | 0.07 |
|  | SAFA | 0.53 | 0.09 | 0.89 | 0.03 | 1.30 | 0.04 |
| Meat | Sodium | 1.16 | 0.02 | 0.83 | 0.05 | 0.49 | 0.08 |
|  | SAFA | 0.99 | 0.02 | 1.06 | 0.02 | 0.91 | 0.03 |
| Sauces | Sugar | 1.11 | 0.04 | 0.87 | 0.04 | 0.96 | 0.04 |
|  | Sodium | -0.57 | 0.41 | 1.15 | 0.09 | 1.14 | 0.06 |
| Snacks | Sugar | 0.96 | 0.03 | 0.76 | 0.04 | 1.13 | 0.02 |
|  | Sodium | 1.07 | 0.02 | 1.10 | 0.03 | 0.63 | 0.04 |
|  | SAFA | 0.80 | 0.03 | 0.69 | 0.04 | 1.16 | 0.02 |

Adjusted for educational level, age, sex ethnicity and household size

SE; Standard Error

Supplementary Table 6. Uncompensated elasticities and corresponding standard errors (shaded boxes showing own price elasticities, others being cross price elasticities)

| **Food categories** | **Nutritional clusters based on sugar content** | | | | **Nutritional clusters based on sodium content** | | | | **Nutritional clusters based on SAFA content** | | | |
| --- | --- | --- | --- | --- | --- | --- | --- | --- | --- | --- | --- | --- |
|  | **Change in quantity** | **Change in price** | | | **Change in quantity** | **Change in price** | | | **Change in quantity** | **Change in price** | | |
|  |  | Low-sugar | Medium-sugar | High-sugar |  | NA | | |  | NA | | |
| Beverages |  | PE (SE) | PE (SE) | PE (SE) |  |  |  |  |  |  |  |  |
|  | Low-sugar | **-1.03** (0.02) | **-0.19** (0.04) | -0.04 (0.02) |  |  |  |  |  |  |  |  |
|  | Medium-sugar | 0.00 (0.01) | **-0.88** (0.03) | -0.01 (0.02) |  |  |  |  |  |  |  |  |
|  | High-sugar | **0.27** (0.11) | -0.25 (0.29) | **-0.52** (0.14) |  |  |  |  |  |  |  |  |
|  |  | Low-sugar | Medium-sugar | High-sugar |  | Low-sodium | Medium-sodium | High-sodium |  | NA | | |
| Grains |  | PE (SE) | PE (SE) | PE (SE) |  | PE (SE) | PE (SE) | PE (SE) |  |  |  |  |
|  | Low-sugar | **-0.96** (0.02) | **-0.07** (0.02) | **-0.08** (0.01) | Low-sodium | **-0.71** (0.04) | **-0.30** (0.05) | 0.01 (0.02) |  |  |  |  |
|  | Medium-sugar | -0.13 (0.11) | **-0.55** (0.10) | 0.05 (0.06) | Medium-sodium | **-0.14** (0.02) | **-0.92** (0.02) | **-0.04** (0.01) |  |  |  |  |
|  | High-sugar | -0.26 (0.14) | 0.05 (0.10) | **-0.30** (0.08) | High-sodium | **0.30** (0.10) | **0.34** (0.14) | **-0.61** (0.11) |  |  |  |  |
|  |  | NA | | |  | Low-sodium | Medium-sodium | High-sodium |  | Low-SAFA | Medium-SAFA | High-SAFA |
| Dairy |  |  |  |  |  | PE (SE) | PE (SE) | PE (SE) |  | PE (SE) | PE (SE) | PE (SE) |
|  |  |  |  |  | Low-sodium | **-0.88** (0.04) | **-0.05** (0.02) | -0.01 (0.02) | Low-SAFA | **-0.98** (0.06) | **0.27** (0.06) | **0.18** (0.06) |
|  |  |  |  |  | Medium-sodium | -0.39 (0.29) | **0.64** (0.24) | 0.35 (0.27) | Medium-SAFA | **0.06** (0.02) | **-0.97** (0.02) | 0.02 (0.02) |
|  |  |  |  |  | High-sodium | **-0.17** (0.07) | **-0.12** (0.04) | **-1.03** (0.05) | High-SAFA | **-0.08** (0.03) | **-0.14** (0.03) | **-1.08** (0.03) |
|  |  | NA | | |  | Low-sodium | Medium-sodium | High-sodium |  | Low-SAFA | Medium-SAFA | High-SAFA |
| Meat |  |  |  |  |  | PE (SE) | PE (SE) | PE (SE) |  | PE (SE) | PE (SE) | PE (SE) |
|  |  |  |  |  | Low-sodium | **-0.95** (0.03) | **-0.15** (0.02) | **-0.07** (0.01) | Low-SAFA | **-0.86** (0.03) | **-0.10** (0.03) | -0.03 (0.02) |
|  |  |  |  |  | Medium-sodium | **-0.18** (0.06) | **-0.71** (0.05) | **0.07** (0.03) | Medium-SAFA | **-0.11** (0.03) | **-0.77** (0.04) | **-0.18** (0.02) |
|  |  |  |  |  | High-sodium | 0.11 (0.09) | **0.19** (0.07) | **-0.79** (0.06) | High-SAFA | -0.02 (0.03) | **-0.23** (0.04) | **-0.66** (0.04) |
|  |  | Low-sugar | Medium-sugar | High-sugar |  | Low-sodium | Medium-sodium | High-sodium |  | NA | | |
| Sauces |  | PE (SE) | PE (SE) | PE (SE) |  | PE (SE) | PE (SE) | PE (SE) |  |  |  |  |
|  | Low-sugar | **-1.08** (0.03) | -0.02 (0.02) | -0.01 (0.03) | Low-sodium | 0.40 (0.35) | 0.18 (0.22) | 0.00 (0.21) |  |  |  |  |
|  | Medium-sugar | 0.06 (0.04) | **-0.88** (0.05) | -0.05 (0.05) | Medium-sodium | **-0.12** (0.05) | **-1.04** (0.06) | 0.01 (0.06) |  |  |  |  |
|  | High-sugar | 0.06 (0.04) | -0.08 (0.04) | **-0.94** (0.06) | High-sodium | **-0.14** (0.05) | 0.01 (0.05) | **-1.01** (0.06) |  |  |  |  |
|  |  | Low-sugar | Medium-sugar | High-sugar |  | Low-sodium | Medium-sodium | High-sodium |  | Low-SAFA | Medium-SAFA | High-SAFA |
| Snacks |  | PE (SE) | PE (SE) | PE (SE) |  | PE (SE) | PE (SE) | PE (SE) |  | PE (SE) | PE (SE) | PE (SE) |
|  | Low-sugar | **-0.93** (0.03) | **0.07** (0.02) | **-0.09** (0.03) | Low-sodium | **-1.01** (0.02) | -0.01 (0.02) | **-0.05** (0.01) | Low-SAFA | **-0.98** (0.04) | **0.08** (0.03) | **0.10** (0.04) |
|  | Medium-sugar | **0.16** (0.04) | **-1.12** (0.03) | **0.20** (0.03) | Medium-sodium | -0.02 (0.02) | **-0.97** (0.03) | **-0.11** (0.02) | Medium-SAFA | **0.11** (0.04) | **-0.99** (0.04) | **0.18** (0.05) |
|  | High-sugar | **-0.12** (0.02) | 0.00 (0.01) | **-1.02** (0.02) | High-sodium | **0.08** (0.03) | -0.05 (0.05) | **-0.66** (0.05) | High-SAFA | **-0.04** (0.01) | **-0.03** (0.01) | **-1.09** (0.02) |

Bold values indicate a p-value < 0.05

Adjusted for educational level, age, sex, ethnicity and household size

NA; Not Applicable

Supplementary Table 7. Compensated elasticities and corresponding standard errors (shaded boxes showing own price elasticities, others being cross price elasticities)

| **Food categories** | **Nutritional clusters based on sugar content** | | | | **Nutritional clusters based on sodium content** | | | | **Nutritional clusters based on SAFA content** | | | |
| --- | --- | --- | --- | --- | --- | --- | --- | --- | --- | --- | --- | --- |
|  | **Change in quantity** | **Change in price** | | | **Change in quantity** | **Change in price** | | | **Change in quantity** | **Change in price** | | |
|  |  | Low-sugar | Medium-sugar | High-sugar |  | NA | | |  | NA | | |
| Beverages |  | PE (SE) | PE (SE) | PE (SE) |  |  |  |  |  |  |  |  |
|  | Low-sugar | **-0.60** (0.02) | **0.59** (0.02) | 0.01 (0.02) |  |  |  |  |  |  |  |  |
|  | Medium-sugar | **0.30** (0.01) | **-0.33** (0.02) | **0.03** (0.01) |  |  |  |  |  |  |  |  |
|  | High-sugar | **0.44** (0.12) | 0.06 (0.18) | **-0.49** (0.14) |  |  |  |  |  |  |  |  |
|  |  | Low-sugar | Medium-sugar | High-sugar |  | Low-sodium | Medium-sodium | High-sodium |  | NA | | |
| Grains |  | PE (SE) | PE (SE) | PE (SE) |  | PE (SE) | PE (SE) | PE (SE) |  |  |  |  |
|  | Low-sugar | **-0.07** (0.02) | **0.06** (0.01) | 0.01 (0.01) | Low-sodium | **-0.45** (0.04) | **0.38** (0.04) | **0.07** (0.02) |  |  |  |  |
|  | Medium-sugar | **0.38** (0.10) | **-0.48** (0.09) | 0.10 (0.06) | Medium-sodium | **0.14** (0.02) | **-0.17** (0.02) | **0.03** (0.01) |  |  |  |  |
|  | High-sugar | 0.14 (0.14) | 0.11 (0.09) | **-0.26** (0.08) | High-sodium | **0.29** (0.09) | **0.32** (0.11) | **-0.61** (0.11) |  |  |  |  |
|  |  | NA | | |  | Low-sodium | Medium-sodium | High-sodium |  | Low-SAFA | Medium-SAFA | High-SAFA |
| Dairy |  |  |  |  |  | PE (SE) | PE (SE) | PE (SE) |  | PE (SE) | PE (SE) | PE (SE) |
|  |  |  |  |  | Low-sodium | **-0.29** (0.03) | -0.01 (0.02) | **0.30** (0.03) | Low-SAFA | **-0.91** (0.05) | **0.52** (0.04) | **0.38** (0.06) |
|  |  |  |  |  | Medium-sodium | **-0.77** (0.25) | **0.61** (0.23) | 0.16 (0.25) | Medium-SAFA | **0.19** (0.01) | **-0.55** (0.02) | **0.36** (0.02) |
|  |  |  |  |  | High-sodium | **0.66** (0.05) | -0.06 (0.04) | **-0.60** (0.06) | High-SAFA | **0.10** (0.02) | **0.47** (0.02) | **-0.57** (0.03) |
|  |  | NA | | |  | Low-sodium | Medium-sodium | High-sodium |  | Low-SAFA | Medium-SAFA | High-SAFA |
| Meat |  |  |  |  |  | PE (SE) | PE (SE) | PE (SE) |  | PE (SE) | PE (SE) | PE (SE) |
|  |  |  |  |  | Low-sodium | **-0.21** (0.03) | **0.14** (0.02) | **0.07** (0.01) | Low-SAFA | **-0.51** (0.03) | **0.30** (0.03) | **0.21** (0.02) |
|  |  |  |  |  | Medium-sodium | **0.34** (0.05) | **-0.50** (0.05) | **0.17** (0.03) | Medium-SAFA | **0.26** (0.03) | **-0.34** (0.03) | **0.08** (0.02) |
|  |  |  |  |  | High-sodium | **0.42** (0.08) | **0.31** (0.06) | **-0.73** (0.06) | High-SAFA | **0.30** (0.03) | **0.13** (0.03) | **-0.44** (0.04) |
|  |  | Low-sugar | Medium-sugar | High-sugar |  | Low-sodium | Medium-sodium | High-sodium |  | NA | | |
| Sauces |  | PE (SE) | PE (SE) | PE (SE) |  | PE (SE) | PE (SE) | PE (SE) |  |  |  |  |
|  | Low-sugar | **-0.60** (0.03) | **0.28** (0.02) | **0.32** (0.03) | Low-sodium | 0.35 (0.33) | -0.08 (0.23) | -0.27 (0.21) |  |  |  |  |
|  | Medium-sugar | **0.43** (0.04) | **-0.64** (0.05) | **0.21** (0.05) | Medium-sodium | -0.02 (0.05) | **-0.52** (0.06) | **0.54** (0.05) |  |  |  |  |
|  | High-sugar | **0.47** (0.04) | **0.19** (0.04) | **-0.65** (0.06) | High-sodium | -0.04 (0.04) | **0.53** (0.05) | **-0.49** (0.05) |  |  |  |  |
|  |  | Low-sugar | Medium-sugar | High-sugar |  | Low-sodium | Medium-sodium | High-sodium |  | Low-SAFA | Medium-SAFA | High-SAFA |
| Snacks |  | PE (SE) | PE (SE) | PE (SE) |  | PE (SE) | PE (SE) | PE (SE) |  | PE (SE) | PE (SE) | PE (SE) |
|  | Low-sugar | **-0.61** (0.03) | **0.25** (0.02) | **0.36** (0.03) | Low-sodium | **-0.56** (0.02) | **0.41** (0.02) | **0.15** (0.01) | Low-SAFA | **-0.81** (0.03) | **0.23** (0.03) | **0.59** (0.03) |
|  | Medium-sugar | **0.41** (0.03) | **-0.97** (0.03) | **0.56** (0.03) | Medium-sodium | **0.44** (0.02) | **-0.53** (0.03) | **0.09** (0.02) | Medium-SAFA | **0.26** (0.04) | **-0.87** (0.04) | **0.61** (0.04) |
|  | High-sugar | **0.26** (0.02) | **0.23** (0.01) | **-0.49** (0.02) | High-sodium | **0.34** (0.03) | **0.20** (0.04) | **-0.54** (0.04) | High-SAFA | **0.20** (0.01) | **0.17** (0.01) | **-0.38** (0.02) |

Bold values indicate a p-value < 0.05

Adjusted for educational level, age, sex, ethnicity and household size

NA; Not Applicable

Supplementary Table 8. Price elasticities and standard errors from the double log model for name- and home-brand products within the food categories

| **Food category** | **Change in quantity** | **Change in price** | | | |
| --- | --- | --- | --- | --- | --- |
|  |  | Name-brand | | Home-brand | |
|  |  | PE | (SE) | PE | (SE) |
| Beverages | Name-brand | **-1.01** | (0.01) | **0.09** | (0.03) |
|  | Home-brand | **0.05** | (0.01) | **-0.89** | (0.02) |
|  |  | Name-brand | | Home-brand | |
| Grains | Name-brand | **-0.80** | (0.03) | -0.05 | (0.03) |
|  | Home-brand | -0.04 | (0.03) | **-0.36** | (0.03) |
|  |  | Name-brand | | Home-brand | |
| Snacks | Name-brand | **-1.00** | (0.05) | 0.03 | (0.03) |
|  | Home-brand | **0.12** | (0.04) | **-1.14** | (0.03) |

Bold values indicate a p-value < 0.05

Adjusted for educational level, age, sex, ethnicity and household size
